# The *Arabidopsis* Mitogen-Activated Protein Kinase Kinase Kinase 20 (MKKK20) Acts Upstream of MKK3 and MPK18 in Two Separate Signaling Pathways Involved in Root Microtubule Functions

**DOI:** 10.3389/fpls.2017.01352

**Published:** 2017-08-08

**Authors:** Rachid Benhamman, Fangwen Bai, Samuel B. Drory, Audrey Loubert-Hudon, Brian Ellis, Daniel P. Matton

**Affiliations:** ^1^Institut de Recherche en Biologie Végétale, Département de Sciences Biologiques, Université de Montréal, Montréal QC, Canada; ^2^Michael Smith Laboratories, University of British Columbia, Vancouver BC, Canada

**Keywords:** MKKK20, MKK3, MPK18, *Arabidopsis*, roots, kinase cascade, microtubules

## Abstract

Mitogen-activated protein kinase (MAPK) signaling networks represent important means of signal transduction in plants and other eukaryotes, controlling intracellular signaling by linking perception of environmental or developmental cues to downstream targets. In the *Arabidopsis* MEKK subfamily, the MKKK19, 20, and 21 form a highly supported clade with the Solanaceous Fertilization-Related Kinases. In *Arabidopsis*, little is known about this group, except for MKKK20, which is involved in osmotic stress. Using a directed MKKK-MKK yeast two-hybrid (Y2H) screen, MKKK20 was found to interact only with MKK3, while a MKKK20 large-scale Y2H screen retrieved MPK18 as a direct interactant. *In vitro* phosphorylation assays showed that MKKK20 phosphorylates both MKK3 and MPK18. However, when all three kinases are combined, no synergistic effect is observed on MPK18 phosphorylation, suggesting a direct access to MPK18, consistent with the absence of interaction between MKK3 and MPK18 in protein–protein interaction assays. Since *mpk18* mutant plants were previously shown to be defective in microtubule-related functions, phenotypes of *mkkk20* single and *mkkk20/mpk18* double mutants were investigated to determine if *MKKK20* acts upstream of *MPK18*. This was the case, as *mkkk20* root length was shorter than WT in media containing microtubule-disrupting drugs as previously observed for *mpk18* plants. Surprisingly, *mkk3* plants were also similarly affected, suggesting the presence of two non-complementary pathways involved in *Arabidopsis* cortical microtubule function, the first including MKKK20, MKK3 and an unknown MPK; the second, a non-canonical MAPK cascade made of MKKK20 and MPK18 that bypasses the need for an MKK intermediate.

## Introduction

Microtubules are polymers of α- and β-tubulin heterodimers that help form the eukaryotic cytoskeleton. They are involved in many physiological processes such as development, morphogenesis, intracellular transport, cell division, and motility. Plant microtubule arrays differ from those in other eukaryotes in that they are dispersed and lack conspicuous organization (Dixit et al., 2006; Hamada, 2014). In addition, plant microtubules are organized into several distinct structures during the cell cycle (Ehrhardt and Shaw, 2006; Yamada and Goshima, 2017). During mitosis and after chromosomal segregation, plant cells build the bipolar phragmoplast array composed mainly of aligned microfilaments and highly dynamic microtubules. These microtubules form parallel arrays in the cell cortex, where they are organized into highly diverse patterns, relying on a variety of mechanisms for orientation, assembly, and function. In interphase cells, microtubules form parallel arrays in the cortex. The orientation of these arrays varies according to the cell type and stage of development. Cortical microtubules, for instance, are perpendicularly oriented during cell elongation, yet form parallel arrays during interphase (Lloyd and Chan, 2008; Chen et al., 2016).

Mitogen-activated protein kinases (MAPKs) were originally shown to phosphorylate the neuronal microtubule-associated protein, MAP2, after stimulation by insulin (Ray and Sturgill, 1987, 1988). Since then, MAPKs have been recognized as one of the most important cellular regulators of biological processes such as differentiation, proliferation, cell death, hormone signaling, osmotic stress, etc. (Colcombet and Hirt, 2008; Lu et al., 2015). Canonical MAPK cascades operate via successive phosphorylation steps starting with an upstream MAPK kinase kinase (MAPKKK or MKKK) that phosphorylates and activates a MAPK kinase (MAPKK or MKK) on one of two serine/threonine residues in a conserved (S/T)-X_5_-(S/T) motif in plants and (S/T)-X_3_-(S/T) in yeast and animals (Garrington and Johnson, 1999; Chang and Karin, 2001). The MKK then phosphorylates a MAPK (or MPK) by dual phosphorylation of the conserved—TXY—motif located in its activation loop. Typical MAPK substrates include transcription factors in addition to other cytoplasmic or nuclear proteins (Rodriguez et al., 2010). Activation of the upstream MAPKKK is more complex, and poorly understood in plants. Studies in yeast and animals show that MAPKKK activation can be mediated by MAPKKK kinases (Elion, 2000), G proteins (Fanger et al., 1997), and G protein-coupled receptors (Sugden and Clerk, 1997) or less frequently by the receiver domain of a two-component histidine kinase complex (Posas and Saito, 1997). The *Arabidopsis thaliana* genome encodes around 80 putative MAPKKKs (including 21 from the MEKK subfamily), 10 MAPKKs (MKKs), and 20 MAPKs (MPKs) (Ichimura and Group, 2002). The plant MAPKKKs can be further classified into two major groups: the MEKKs subgroup containing 21 kinases similar to animal MEKKs and yeast MAPKKKs, and the Raf-like kinases. Phylogenetically close sister clades such as the ZIK and MLK-like kinases are also considered as MAPKKKs although, so far, only the MEKKs are involved in MAPK cascades (Rodriguez et al., 2010).These kinases have been implicated in a wide array of plant biological processes ranging from stress responses to development (Rodriguez et al., 2010). To date, however, only a limited number of complete MAPK cascades have been reported in plants, mainly in abiotic stress responses, plant–pathogen interactions and in plant development. For example, the first completely identified MAPK cascade was MEKK1-MKK4/MKK5-MPK3/MPK6, which acts upstream of two WRKY transcription factors, WRKY22/WRKY29. This cascade functions downstream from the FLS2 flagellin receptor kinase that ultimately enhance resistance and protects against multiple bacterial and fungal pathogens (Asai et al., 2002). In plant development, the *A. thaliana* MAPKKK YDA acts upstream of the MKK4/MKK5-MPK3/MPK6 signaling module, which functions in stomatal patterning and inflorescence development (Wang et al., 2007; Meng et al., 2012). In biotic and abiotic stress responses, the *Nicotiana benthamiana* MAPK cascade NPK1-MEK1-Ntf6 is involved in tobacco mosaic virus (TMV) resistance (Jin et al., 2002; Liu et al., 2004), while in *A. thaliana*, the MEKK1-MKK2-MPK4/MPK6 cascade mediates cold and salt stress responses (Teige et al., 2004), and the MEKK1-MKK1/MKK2-MPK4 cascade functions as a negative regulator in innate immune responses (Gao et al., 2008). More recently, some ABA-regulated abiotic stress genes where also shown to depend on the MAPKKK17/18-MKK3-MPK1/2/7/14 cascade (Danquah et al., 2015).

The role of MAPK signaling in regulating microtubule functions has been widely documented in animals (Hoshi et al., 1992; Mandelkow et al., 1995; Verlhac et al., 1996; Terret et al., 2003; Zhao and Chen, 2006; Liu et al., 2007; Sun et al., 2008). Meanwhile, the involvement of MAPK-signaling in the regulation of plant microtubule functions has begun to emerge. In *Arabidopsis*, inhibition of protein tyrosine kinases and tyrosine phosphatases was reported to affect the dynamics and organization of cortical microtubules (Yemets et al., 2008). *At*MPK4 and *At*MPK6 were also shown to be necessary for cell division and localize to the phragmoplast during cytokinesis (Beck et al., 2011; Komis et al., 2011). Phosphorylation of the tobacco microtubule-associated protein MAP65-1 by the MAPK NRK1/NTF6 down-regulates the microtubule-bundling activity of NtMAP65-1 and positively regulates expansion of the phragmoplast (Sasabe et al., 2006). In addition, the NtMAP65-1 *Arabidopsis* ortholog, *At*MAP65-1, has been shown to bind microtubule bundles specifically during interphase, anaphase, and telophase. When ectopically expressed in tobacco cells, a non-phosphorylatable *At*MAP65-1 version affects mitotic division (Smertenko et al., 2006).

*Arabidopsis* MKKK20 participates in the osmotic stress response through its regulation of MPK6 (Kim et al., 2012), and was shown to interact with calmodulins (CaM) and CaM-like proteins (CML) in protein array assays (Popescu et al., 2007). Recently, insertional mutants of MKKK20 showed insensitivity to ABA in terms of root growth and stomatal response. In this context, MKKK20 was demonstrated to modulates abscisic acid responses acting upstream of MKK5 and MPK6 (Li et al., 2017). *Arabidopsis* MAPKKs have been classified into four groups, A–D (Soyano et al., 2003; Teige et al., 2004; Dai et al., 2006), with *At*MKK3 belonging to group B, a single-member clade characterized by the presence of a C-terminal NTF2 domain known to promote nuclear import of cargo proteins (Steggerda and Paschal, 2002). This feature could confer to MKK3 an important and singular rule among all other AtMKKs (Colcombet et al., 2016). MKK3 participates in pathogen signaling and is required for regulation during dark–light transition (Doczi et al., 2007; Lee, 2015). *Arabidopsis* root growth inhibition by jasmonate is negatively regulated by the MKK3/MPK6 cascade (Takahashi et al., 2007). This cascade, along with MYC2, is involved in blue light-mediated seedling development in *Arabidopsis* (Sethi et al., 2014). MKK3 also participates in an abscisic acid-activated MAPK cascade (Danquah et al., 2015). Recently, two MKK3 orthologs have been found to regulate seed dormancy in barley (*Hordeum vulgare* L.) and bread wheat (*Triticum aestivum* L.) (Nakamura et al., 2016; Torada et al., 2016). MAPKs are also divided into four groups (Ichimura and Group, 2002). *At*MPK18 belongs to group D, whose members harbor a—TDY—motif in their activation loop whereas the three other groups have a—TEY—signature. *At*MPK18 had been reported to interact with the phosphatase propyzamide hypersensitive 1 (PHS1) and is involved in microtubule functions (Walia et al., 2009).

Here we show that *Arabidopsis* MKKK20 physically interacts with both MKK3 and MPK18 and phosphorylates these kinases *in vitro*. Surprisingly, phosphorylated MKK3 did not increase MPK18 phosphorylation status when the three kinases were put together. Previous analysis showed that MPK18 is involved in microtubule-related functions in *Arabidopsis* (Walia et al., 2009). When tested in the presence of microtubule depolymerizing drugs, *mkkk20* and *mkk3* mutant plants were found to have shorter roots than wild type, resembling the *mpk18* behavior. This, combined with the analysis of *mkkk20*/*mpk18* and *mkk3/mpk18* double mutants suggests that MKKK20 modulates cortical microtubule functions through two independent MAPK signaling cascades, including a non-canonical pathway that bypasses the need for an intermediary MKK.

## Materials and Methods

### Plant Materials, Growth conditions, and Inhibitor Treatments

*Arabidopsis* wild type (Col-0 accession), *MKKK*20 (SALK_021755 and SALK_124398), *MPK18* (SALK_069399), and *MKK3* (SALK_051970) T-DNA mutants were obtained from TAIR^1^ (for T-DNA line details, see **Supplementary Table S1**). Seeds were grown in growth cabinets at 21°C with a light/dark cycle of 16 h/8 h photoperiod. Primers used to screen for homozygous lines are listed in **Supplementary Table S2**. For root growth assays (elongation rate and skewing angles), seeds were grown aseptically on Hoagland medium solidified with 1.2% agar. For oryzalin (Sigma–Aldrich) treatments, small volumes of a concentrated stock solution in DMSO were added to the molten agar; controls received the same amount of DMSO. Seeds sown on agar plates with or without oryzalin were kept for at least 7 days and placed vertically in a growth cabinet (21°C, light/dark cycle of 16 h/8 h and 120 μmol m^-2^ s^-1^).

### Yeast Two-Hybrid Screening

For large-scale yeast two-hybrid (Y2H) screening, a normalized library made from 11 *Arabidopsis* tissues was used (Mate and Plate Library - Universal *Arabidopsis* Clontech^®^). The library was transformed into yeast strain Y187 (MATα), which can be readily mated to a MATa GAL4 reporter strain. The Matchmaker Gold Yeast Two-Hybrid System (Clontech^®^) was then used to screen the library. The *MKKK20* cDNA bait was cloned into the pGBKT7 vector (GAL4 DNA-BD) and transformed into the Y2H Gold yeast strain (Clontech^®^). Mating of the two sexually different strains was carried out at 30°C for 24 h and the resulting zygotes were plated directly on high stringency SD growth medium (–Leu/–Trp/–Ade/–His) supplemented with 40 μg/ml X-α-Gal and 125 ng/ml Aureobasidin A. DNA from blue yeast colonies that survived this stringent selection was extracted and sequenced using the pGADT7-T 5AD primers. For the directed pairwise Y2H screen, the ProQuest^TM^ Two-hybrid system from Invitrogen was used. All kinases were cloned and sequence-verified before being transferred into Gateway-compatible destination vectors to generate the bait vector *MKKK20* (in pDEST32) as the GAL4 DNA-binding domain (DBD) and the prey vectors *AtMKK1–10* (in pDEST22) as the GAL4-activating domain (AD) and introduced pairwise into the yeast strain MaV203. Controls provided were used according to the manufacturer’s instructions. Interaction strength for HIS3 and URA3 activation assays were scored by visual comparison to the controls.

### Promoter-GUS Expression and Microscopic Observations

For GUS expression, a 1.5-kb promoter region upstream of the MKKK20 coding sequence was cloned into the pMDC162 Gateway^TM^ vector harboring the β-glucuronidase gene to generate an N-terminal fusion reporter gene (Curtis and Grossniklaus, 2003). Primers are listed in **Supplementary Table S3**. The recombinant plasmid was then transformed into *Agrobacterium tumefaciens* C56C1 strain and used to transform wild type *Arabidopsis* (Col-0 accession) plants by the floral dipping method as described previously (Zhang et al., 2006). Seeds from M1 transformed plants were collected and grown in MS media containing 15 μg/ml hygromycin B for selection as described (Harrison et al., 2006). The M1 generation was analyzed for GUS gene expression and the true transformants (strongly growing seedling with well-developed hypocotyls) were grown on to generate an M2 generation. GUS staining was conducted as described before (Weigel and Glazebrook, 2002). Freshly harvested tissues from independent plant lines were collected and kept in 80% acetone before performing the β-glucuronidase assays in 100 mM potassium phosphate buffer, pH 7, 0.1% Triton X-100, 5 mM K_3_Fe(CN)_6_ and 5 mM K_4_Fe(CN)_6_. Tissues were then incubated in 2 mM X-Gluc at 37°C for 4 h. The plant material was then cleared with increasing ethanol concentrations from 20 to 70%, for 30 min each. Observations were performed using a Discovery V12 stereomicroscope or a Zeiss Axio Imager 1 microscope (under bright or dark field) and images were captured using an AxiocamHRc camera (Zeiss, Canada). Clearing of flowers and seedlings was performed by incubating tissues in 100% ethanol for 1 h before transferring them to a methyl salicylate/EtOH solution with increasing ratios (1:3, 1:1, and 3:1) of methyl salicylate for 30 min each. The tissues were subsequently kept in 100% methyl salicylate (Sigma–Aldrich) at 4°C for no more than 24 h to avoid discoloration. The slides were mounted with 100% methyl salicylate and the observations were performed using a Zeiss Axio Imager 1 microscope under bright field.

### RNA Isolation for RT-PCR

For RT-PCR, total RNA from various plant tissues was isolated using the TRIzol^®^ RNA isolation reagent (Life Technologies) according to the manufacturer’s instructions. Reverse transcription was performed using a first-strand cDNA synthesis M-MLV RT kit (Invitrogen). RT-PCR was conducted with gene-specific primers (**Supplementary Table S3**).

### GFP and Bimolecular Fluorescence Complementation Assays

*MKKK20*, *MKK3*, and *MPK18* full-length cDNAs were PCR-amplified, cloned into the Gateway entry vector pDONR^TM^/Zeo (Invitrogen) and sub-cloned into pMCD83 to generate an N-terminal fusion with the GFP marker, or into the plant bimolecular fluorescence complementation (BiFC) vectors pUC-SPYCE or pUC-SPYNE (Walter et al., 2004). All GFP and BiFC constructs were sequence-verified and prepared for transient transformation of onion epidermal cells by microparticle bombardment using a Bio-Rad PDS-1000/He bombardment system as described previously (Germain et al., 2008). The *Arabidopsis* bZIP63 transcription factor was used as positive control in BiFC assays (Walter et al., 2004).

### *In Vitro* Kinase Assays

*MKKK20*, *MKKK20*-KD (kinase dead), and *MPK18* were expressed as His-tag fusion proteins in the pQLinHD (Addgene^®^)^2^ and *MKK3* was expressed as a His-GST-tag in the pDEST-565 vector (Addgene^®^)^3^ using recombinant modified primers (see **Supplementary Table S4**). Plasmids were transformed in the Rosetta-gami *Escherichia coli* strain. MKKK20-KD was generated by replacing the conserved lysine (K) in the kinase ATP-binding loop by a methionine (M). Bacterial cultures in LB media were induced with 0.1 mM isopropyl-1-thio-β-D-galactopyranoside (IPTG) after reaching an OD_600_ absorbance of 0.5 and incubated at 37°C for an additional 3 h (except for MPK18; 25°C overnight). Bacterial cells were pelleted at 10,000 rpm for 10 min and purified under denaturing conditions by re-suspending bacteria in 10 ml lysis buffer (100 mM NaH_2_PO_4_, 10 mM Tris–Cl, 8 M urea, 1 mM PMSF, pH 8.0) per 100 ml. Bacterial cells were disrupted (twice) using a French pressure cell press at 950 psi. The resulting mixture was centrifuged at 10,000 rpm for 10 min and the supernatant collected. Recombinant proteins were purified on HisTrap Ni columns using an ÄKTA Avant FPLC (GE Healthcare). For all kinase assay combinations, 1 μg of each His-kinase was incubated for 30 min in 30 μl kinase reaction buffer containing (50 mM Tris–HCl, pH 7.5, 1 mM DTT, 10 mM MgCl_2_, 10 mM MnCl_2_, 50 mM ATP, and 0.037 MBq [γ ^32^P] ATP. Five micrograms of myelin basic protein (MBP) was added when indicated and enzymatic reactions were stopped by adding SDS sample buffer and heating for 4 min at 95°C. Assays were then fractionated in an SDS-PAGE gel, stained by Coomassie Brilliant Blue and autoradiographed using a Typhoon Trio phosphorimager (GE Healthcare).

### Immunoblotting

Proteins were separated on SDS/PAGE (12% gel) and subsequently blotted onto a nitrocellulose membrane (Millipore 0.2 μM) and blocked in TBS containing 3% dried non-fat skimmed milk powder, pH 7.4 solution for 2 h. An anti-p-ERK antibody (phospho-ERK; Cell Signaling Technology) was used as first antibody and the HRP conjugated Goat anti-Rabbit IgG (H+L) secondary antibody (Thermo Fisher Scientific) as the second antibody.

### Complementation Test and Transgenic Transformation

*MKKK20* and *MKK3* cDNAs were PCR-generated using modified primers (**Supplementary Table S5**) that included a 6x-His tag addition to the protein C-terminal region and cloned in the pDONR^TM^/Zeo vector (Invitrogen). The constructs were then transferred into the Gateway^®^ binary vector pMDC32 (Curtis and Grossniklaus, 2003) to produce the *p35S*::*MKKK20-6xHis* and *p35S*::*MKK3-6xHis* vectors. These constructs were used to complement *mkkk20-1* and *mkk3* mutant lines, respectively, using the floral dip method as described previously (Zhang et al., 2006). Positively transformed lines were selected using the above-mentioned protocol (Harrison et al., 2006) and confirmed by western blotting using an anti-His antibody (**Figure 6**).

## Results

### MKKK20 Yeast-Two Hybrid Interactome

Two Y2H screens were performed to identify possible MKKK20 interacting proteins and candidate substrates. First, a global Y2H screen was performed using MKKK20 as bait against a normalized *A. thaliana* cDNA library (prey library) made from 11 tissues, including etiolated seedlings, seedlings with roots, open flowers, flower buds from different stages, pollen, siliques from all stages, stems and leaves. A two-step screen was performed. After mating, diploid yeast cells were selected based on their ability to grow on a -Leu/-Trp media that also contained Aureobasidine A (AbA), an antibiotic toxic to yeast (*AUR1-C* gene reporter) and X-α-Gal (*MEL1* gene reporter). Both reporters are activated in response to two-hybrid interactions. This led to the isolation of 422 blue colonies. These colonies were then grown on a medium that used the four available reporters simultaneously (*HIS3*, *ADE2*, *AUR1-C*, *MEL1*). This further decreased the number of MKKK20 putative interactants to 66 non-redundant candidates. Interestingly, gene ontology analysis revealed that the GO term signaling (GO:0023052) counted for a third of the MKKK20 interacting proteins (**Supplementary Table S6; S6.1** Process: biological process annotation level 2). These included seven protein kinases (five RLKs and one RLCK), one ARM-repeat kinase and a MAPK, the MPK18. Two protein phosphatases and two kinase binding protein were also retrieved. Also amongst the most represented categories, 10 are involved in cytoskeletal and cell wall functions and eight were transcription factors.

### MKKK20 Interacts Directly with MKK3 and MPK18

**FIGURE 1:**
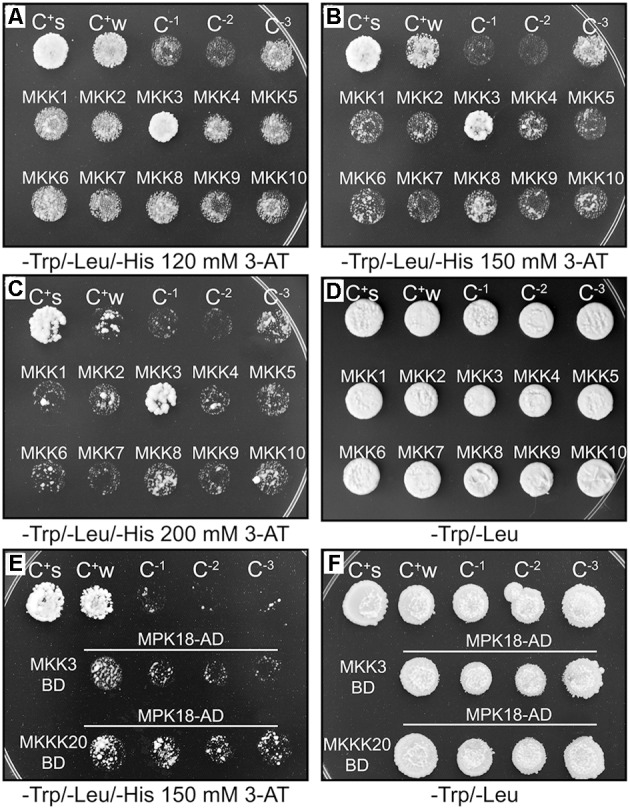
MKKK20/AtMKKs and MKK3/MPK18 yeast two-hybrid interactions. **(A–D)** MKKK20/AtMKKs interaction screen. MKKK20 interacts strongly with MKK3. All kinases were cloned and sequence-verified prior to their transfer into Gateway^TM^ yeast two-hybrid bait and prey vectors (pDEST32 and pDEST22 from Invitrogen^TM^). The clones, MKKK20 (in pDEST32 vector) as GAL4 DNA-binding domain (DBD) and *At*MKKs (in pDEST22 vector) as GAL4-activating domain (AD), were introduced pairwise into the yeast strain MaV203. Interaction strength for HIS3 and URA3 activation assays was scored visually by comparing to the controls. C^+s^: pEXP^TM^32/Krev1/pEXP^TM^22/RalGDS-wt as strong positive interaction control. C^+w^: pEXP^TM^32/Krev1/pEXP^TM^22/RalGDS-m1 as a weak positive interaction control. C^-1^: pEXP^TM^32/Krev1 pEXP^TM^22/RalGDS-m2 as a negative interaction control. C^-2^: pDEST^TM^32/pDEST^TM^22 as a negative activation control. C^-3^: pDEST^TM^MKKK20/pDEST^TM^22 as a negative activation control. **(E,F)**. MKKK20 and MKK3 interaction with MPK18. MKK3 cloned as DBD and MPK18 as AD. The same controls were used as above except C^-2^ served as a negative activation control for MKK3 and C^-2^ as a negative activation control for MPK18.

**FIGURE 2:**
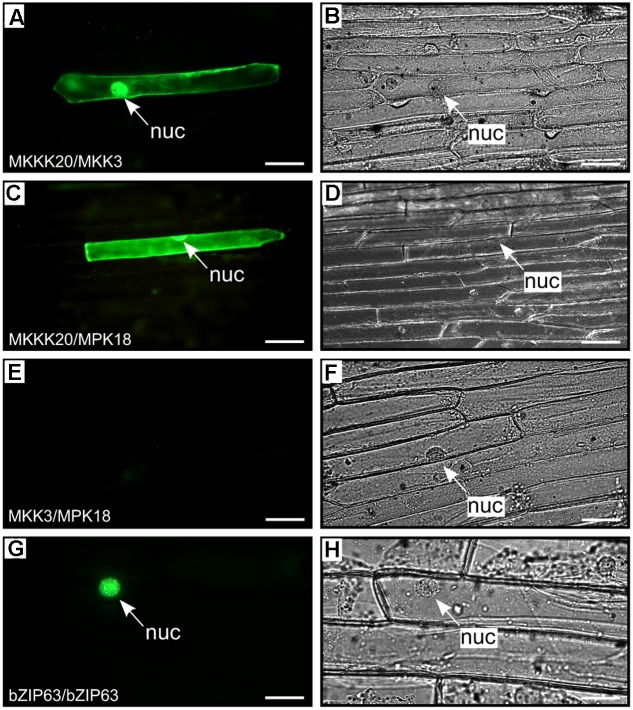
BiFC kinase interaction assays. BiFC visualization of MKKK20 dimerization with MKK3 and MPK18 in onion epidermal cells. Fluorescent **(A,C,E,G)** and bright field **(B,D,F,H)** images of epidermal leaf onion cells cotransformed by vectors harboring split YFP and different kinase pairs: MKKK20/MKK3 **(A,B)**; MKKK20/MPK18 **(C,D)**; MKK3/MPK18 **(E,F)**. Microparticle bombardment was observed under a fluorescence microscope. Full-length cDNAs from the three kinases were cloned in-frame with a split-YFP construct from either BiFC vector pUC-SPYCE or pUC-SPYNE (Walter et al., 2004). The bZIP63 transcription factor was used as positive control for BiFC **(G,H)**. Scale bar = 20 μm.

In a canonical MAPK cascade, sequential phosphorylation and activation events activate the kinase members of the module. Thus, the upstream MKKK phosphorylates and activates the MKK, which in turn phosphorylates and activates the MPK. The absence of a MKK interactor in our MKKK20 global Y2H screen prompted us to screen all 10 *Arabidopsis* MKKs against MKKK20. In a directed pairwise Y2H screen, only MKK3 was found to strongly interact with MKKK20 under stringent conditions (**Figures 1A–C**). Although MKKK20 physically interacted with MPK18 in the previous Y2H systems, MKK3 might also interact with MPK18, as would be expected in a canonical MAPK cascade. However, the pairwise Y2H interaction between MKK3 and MPK18 was much weaker than that detected between MKKK20 and MKK3 (**Figure 1E**). To confirm the physical association observed in the Y2H assays between MKKK20, MKK3, and MPK18, BiFC assays were performed in onion leaf epidermal cells. A clear YFP signal was detected in both the nucleus and the cytoplasm when MKKK20 and MKK3 were bombarded together (**Figures 2A,B**), as well as for MKKK20 and MPK18 (**Figures 2C,D**). However, no signal was detected with the MKK3-MPK18 BiFC assay (**Figures 2E,F**), consistent with the interaction results obtained from the Y2H screen.

### Expression Pattern of MKKK20, MKK3, and MPK18

**FIGURE 3:**
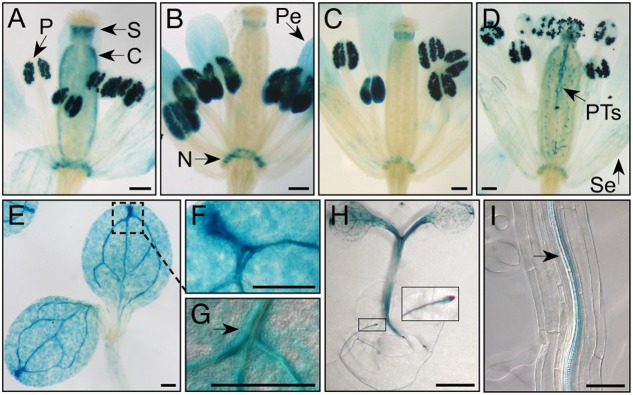
MKKK20 expression pattern monitored by the GUS reporter gene. GUS reporter gene expression under the control of the *MKKK20* promoter in flower buds and seedlings. **(A–D)** Flower developmental stages. **(A)** Stage 11. **(B)** Stage 12. **(C)** Stage 13. **(D)** Stage 14 (according to Smyth et al., 1990). Strong staining is observed in pollen of maturing flowers. Expression tends to become stronger between stage 11 **(A)** and at anthesis **(D)**. Global visualization of flower development, after manual opening, confirmed that pollen grains (P) exhibit strong *MKKK20* expression, in addition to carpel (C), style (S), nectaries (N), petals (Pe), and sepals (Se), the two later with lesser intensities as they mature **(A–D)**. **(D)** While pollen expression last after pollination in pollen tube (PT), staining decreases in the others flower organs. Expression can be detected also in some epidermal cells and vascular tissues. **(E–G)** Expression in cotyledons. **(H)** Expression in hypocotyl and roots. **(I)** Expression in companion cells of phloem tissues of roots. Scale bar: 200 μm, except for **(H)** (5 mm) and **(I)** (100 μm).

**FIGURE 4:**
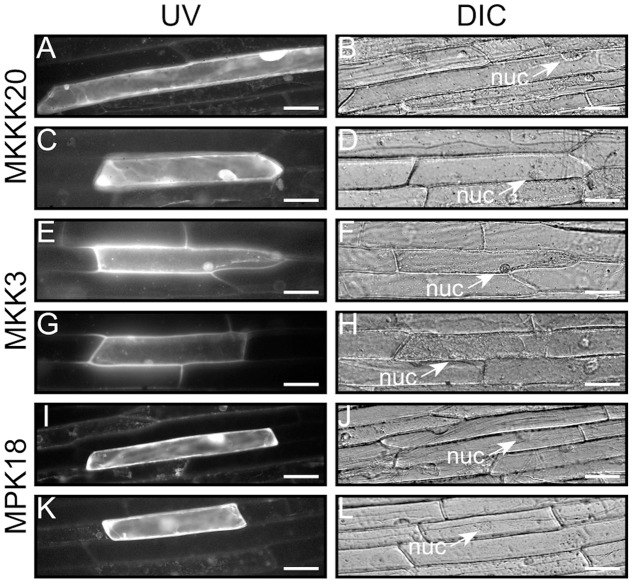
Kinase subcellular localization. Characterization of the MKKK20, MKK3, and MPK18 nuclear and cytosolic localization. The coding regions of the three kinases were fused in-frame to the N-terminus of the GFP reporter in the pMDC83 vector (Curtis and Grossniklaus, 2003). Epifluorescence **(A,C,E,G,I,K)** and bright field **(B,D,F,H,J,L)** images of onion leaf epidermal cells show cytosolic and nuclear localization for all three kinases. Scale bar: 50 μm.

Semi-quantitative RT-PCR analyses from various *Arabidopsis* tissues were conducted to determine the extent of co-expression of *MKKK20*, *MKK3*, and *MPK18*. **Supplementary Figure S1** shows that all three kinases were ubiquitously expressed in the selected tissues, albeit to different degrees. This was confirmed through bioinformatics analyses from publicly available microarray data resources^4^ (BAR^5^), which showed that, under normal conditions, all three kinases are weakly expressed in all tissues (data not shown). *MPK18* and *MKK3* tissue-specific gene expression was previously investigated using histochemical analysis with the GUS reporter gene under the control of their respective promoters. GUS expression was detected in all major tissue types for *MPK18* including roots, leaf vasculature, guard cells and stigma (Walia et al., 2009). Likewise, *MKK3* was detected in all major tissues (stems, leaves, roots, flowers), with higher GUS activity in the vasculature, stipules, nectaries, and root tips (Lampard, 2006). For *MKKK20*, transgenic plants were generated to express the GUS reporter gene under the control of a 1.5-kb promoter sequence upstream of the *MKKK20* translation start site. Visualization of GUS expression during flower development revealed strong expression in pollen grains increasing from young buds to later stages of maturation (**Figures 3A–D**), more specifically stages 11–14. *MKKK20* is expressed to a lesser extent in the gynoecium, particularly in the style and carpel, but not in the stigma regardless of its developmental stage. *MKKK20* is also expressed in nectaries, petals, and sepals (**Figures 3A–D**). Expression was also detected in pollen tubes (**Figure 3D**). Expression was also remarkably strong in seedling leaves (**Figures 3E–G**) and roots (**Figures 3H,I**). Subcellular localization of the three kinases was also examined. Under control of the CaMV 35S promoter, *MKKK20*, *MKK3*, and *MPK18* coding sequences were fused N-terminal to the GFP reporter gene and examined following transient expression in onion leaf epidermal cells using microparticle bombardment. All three kinases were observed both in the cytoplasm and the nucleus (**Figure 4**), a subcellular localization pattern identical to that determined previously through BiFC interactions (**Figure 2**). Prediction models based on more than 10 algorithms for each kinase, combined with published curated data sets from the subcellular location database for *Arabidopsis* proteins (SUBA4^6^) also gave equally nuclear/cytoplasmic localization predictions, in line with the previous subcellular localization assays (**Figures 2**, **4**).

### MKKK20 Autophosphorylates and Phosphorylates Both MKK3 and MPK18 *In Vitro*

**FIGURE 5:**
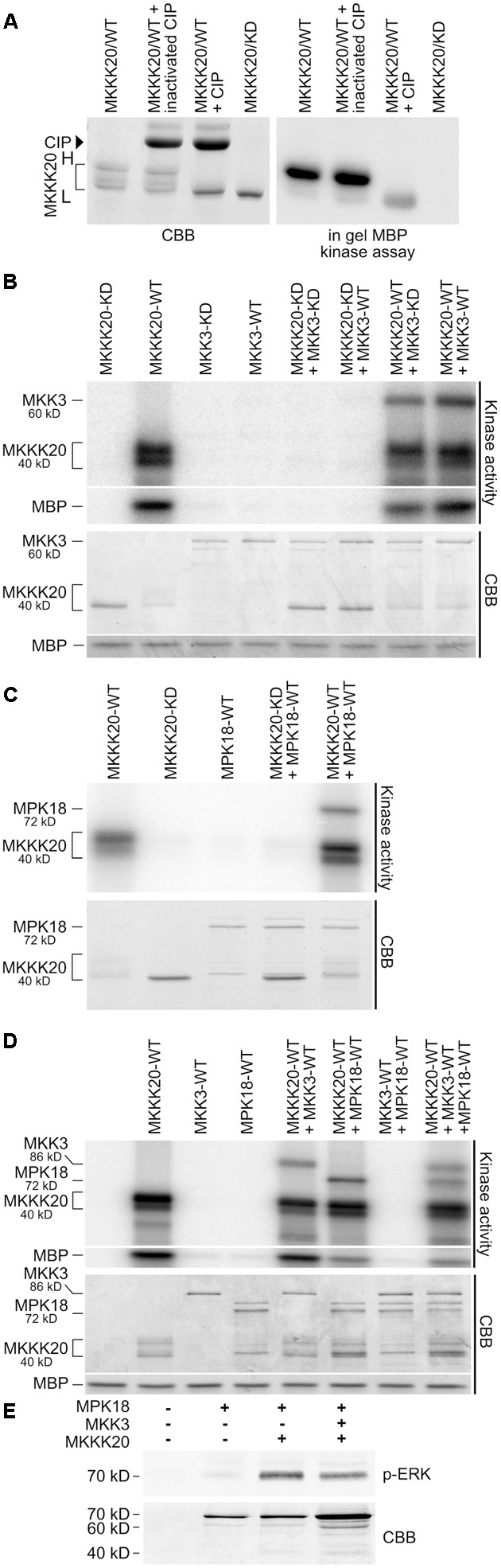
MKKK20 functions upstream of both MKK3 and MPK18. **(A)** MKKK20 phosphorylation affects electrophoretic mobility and kinase activity. Bacterially expressed MKKK20 shows the presence of multiple bands with two major MKKK20 isoforms (see brackets in panels **A–D**; H and L, higher and lower MW isoforms, respectively). Left panel: Coomassie Brilliant Blue (CBB) SDS-PAGE gel of WT MKKK20 treated with active or inactivated calf intestinal alkaline phosphatase (CIP) and an MKKK20 kinase dead version. Right panel: In-gel kinase assay with MBP (0.5 mg/ml) as substrate. *In vitro* kinase assay of recombinant MKKK20/MKKK20^KD^ activity with or without MKK3 **(B)**, or MPK18 **(C)**, and with the three kinases combined **(D)**. All 6xHis-tagged proteins were expressed in bacteria and purified on Ni-NTA Sepharose columns. Two different MKK3 constructs were used. In **(B)** 6xHis-MKK3 and in **(D)** 6xHis-GST-MKK3. Protein combinations were incubated in kinase assay buffer with [γ -^32^P]-ATP. MBP was also added in the *in vitro* kinase assays of panels **(B,D)** as a universal substrate to assess kinase activity. The bottom panel shows the Coomassie Brilliant Blue-stained SDS/PAGE. WT, wild type kinase; KD, kinase dead; CBB, Coomassie Brilliant Blue. **(E)** MPK18 activation loop phosphorylation by MKKK20 as observed with an anti-p-ERK antibody specifically targeting the [T-X-Y] motif.

Full-length MKKK20, MKK3, and MPK18 cDNAs were cloned and expressed as His-tag or His-GST-tag proteins in an *E. coli* system. Kinase activity was measured from *in vitro* kinase assays and in-gel kinase assays. Firstly, kinase autophosphorylation activity and ability to phosphorylate the MBP generic substrate were tested. Unlike MKK3 (**Figures 5B,D**) and MPK18 (**Figures 5C,D**) proteins, MKKK20 strongly autophosphorylates (**Figures 5A–D**) and phosphorylates MBP (**Figures 5A,B,D**). Interestingly, migration on SDS-PAGE gels of bacterially expressed MKKK20 revealed the presence of multiple bands with two major MKKK20 isoforms (higher-H and lower-L isoforms; **Figures 5A–D**). Differences in phosphorylated residues could explain the migration shift of the various MKKK20 phosphoisoforms in SDS PAGE gels. To test this, MKKK20 was incubated with active or inactivated calf intestinal alkaline phosphatase (CIP) followed by an in-gel kinase assay with MBP as substrate (**Figure 5A**). Dephosphorylation of MKKK20 as well as its kinase dead version (**Figure 5A**) led to a single MW isoform (lower MW), confirming that the multi-band pattern from the bacterially expressed MKKK20 is due to differential levels of phosphorylation among isoforms. MKKK20 kinase activity is also heavily dependent on its phosphorylation status since dephosphorylation strongly weakened its activity toward MBP (**Figure 5A**). Furthermore, the MKKK20 higher MW isoform consistently exhibited higher autophosphorylation activity (**Figures 5A–D**) and greatly increased activity toward the generic substrate MBP (**Figure 5A**, right panel). Mass spectroscopy analysis of the high and low molecular weight isoforms of MKKK20 revealed differences in phosphorylation sites (**Supplementary Figure S2** and **Table S7**). Six phosphorylated amino acids were unique to HMW or LMW MKKK20/WT protein. Of these, two phosphorylated tyrosine were unique to the MKKK20-HMW. From mass spectrometry data, both MKKK20-HMW and MKKK20-LMW displayed around 20 phosphorylated sites from peptides that covered 40% of the whole protein. This represents half of the total peptides detected (phosphorylated and non-phosphorylated), which covered 80% of the complete protein. MKKK20 phosphorylated sites from the high and low molecular weight variants were solely found in the kinase domain, while only a single phosphoserine was found in the kinase dead version of MKKK20, outside of the kinase domain. Next, MKKK20 was incubated with MKK3 and MPK18, separately and in combinations. As expected from the tight interaction between MKKK20 and MKK3 observed in the Y2H screen, MKKK20 strongly phosphorylated MKK3-WT as well as MKK3-KD, while MKK3-WT and MKK3-KD alone or in the presence of MKKK20-KD neither autophosphorylated nor phosphorylated MBP (**Figure 5B**). Since MPK18 was also found to directly interact with MKKK20 in the Y2H screen (**Figure 1E**), the ability of MKKK20 to phosphorylate MPK18 was also tested. MPK18 alone did not display autophosphorylation activity but was a good substrate for the MKKK20 (**Figures 5C,D**), confirming the direct interaction with these two kinases, as shown previously from Y2H and BiFC assays. When incubated together, MKK3 and MPK18 did not show any reciprocal kinase activity nor did they phosphorylate MBP (**Figure 5D**). This could suggest that MKK3 needs to be activated by the upstream MKKK20 to phosphorylate the downstream MPK18. However, when the three kinases were incubated together, no synergistic phosphorylation effect was observed on MPK18, suggesting that phosphorylated MKK3 does not phosphorylates MPK18 (**Figure 5D**). In fact, MKKK20 displayed no obvious phosphorylation preference for either MKK3 or MPK18, both kinases being equally phosphorylated by the MKKK20 (**Figure 5D**). To further ascertain that MKKK20 directly activates MPK18 and bypass the need of an MKK, an anti-p-ERK antibody that specifically targets the [T-X-Y] MPK activation loop was used. **Figure 5E** shows that a combination of MKKK20 and MPK18 is sufficient to phosphorylate the MPK18 activation loop TDY motif. Furthermore, when all three kinases are combined, no synergistic effect on MPK18 is found, as observed in the previous kinase assays (**Figure 5D**), confirming that MKKK20 bypasses the need of an MKK to activate MPK18.

### *mkkk20* and *mkk3* Mutants Are Sensitive to Microtubule-Disrupting Drugs

**FIGURE 6:**
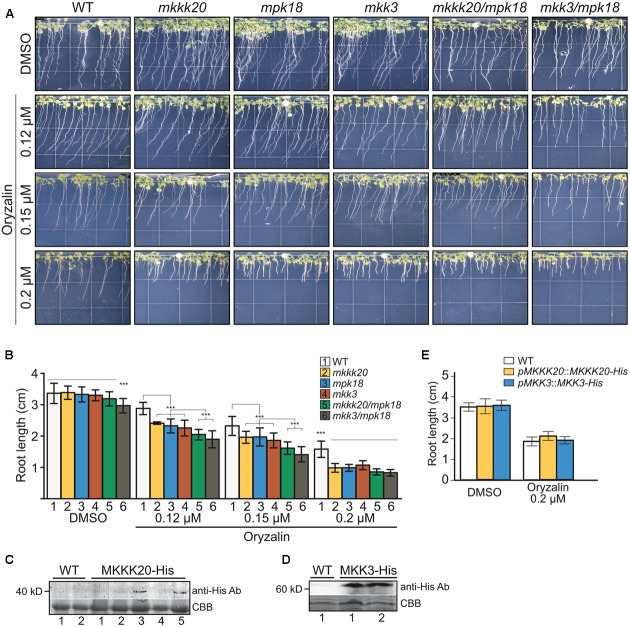
Absence of *MKKK20* and *MKK3* affects microtubule-related functions. Root length of 8-day-old wild type, single mutant *mkkk20-1*, *mkk3*, *mpk18*, and double mutant *mkkk20/mpk18*, *mkk3/mpk18* seedlings grown on DMSO and various concentrations of oryzalin. **(A)** Whole seedlings were grown vertically in Hoagland medium solidified with 1.2% agar with different oryzalin concentrations. Root length and skewing angle are shown. **(B)** Treatments with 0.12, 0.15, and 0.2 μM oryzalin significantly reduced the length of all mutant roots compared to the wild type, based on one-way ANOVA multiple comparison with Tukey’s test. ^∗∗∗^*p* < 0.001 for all treatments. Data represent means ± SD (*n* ≥ 30). Significant reductions, if any, in root length between single and double mutants are also indicated. **(C–E)** MKKK20 and MKK3 complementation. Western blot analyses of His-Tag proteins showing expression of the recombinant kinases in their respective mutant backgrounds MKKK20 **(C)** and MKK3 **(D)**. Plants 3 and 5 for MKKK20 and samples 1 and 2 for MKK3 were kept to carry out further plants analyses. Upper panel in **(C)** and **(D)**. Detection of His-Tag proteins was done with a primary mouse anti-poly-histidine antibody, followed by a secondary rabbit anti-mouse IgG-HRP antibody for colorimetric detection. Lower panel in **(C)** and **(D)**. CBB staining of total protein. **(E)** Root length of 6-day-old *mkkk20* and *mkk3* complemented seedlings grown on DMSO and 0.2 μM oryzalin. Data represent means ± SD (*n* ≥ 28). For the effect of oryzalin on root length in mutants, refer to panel **(B)**.

**FIGURE 7:**
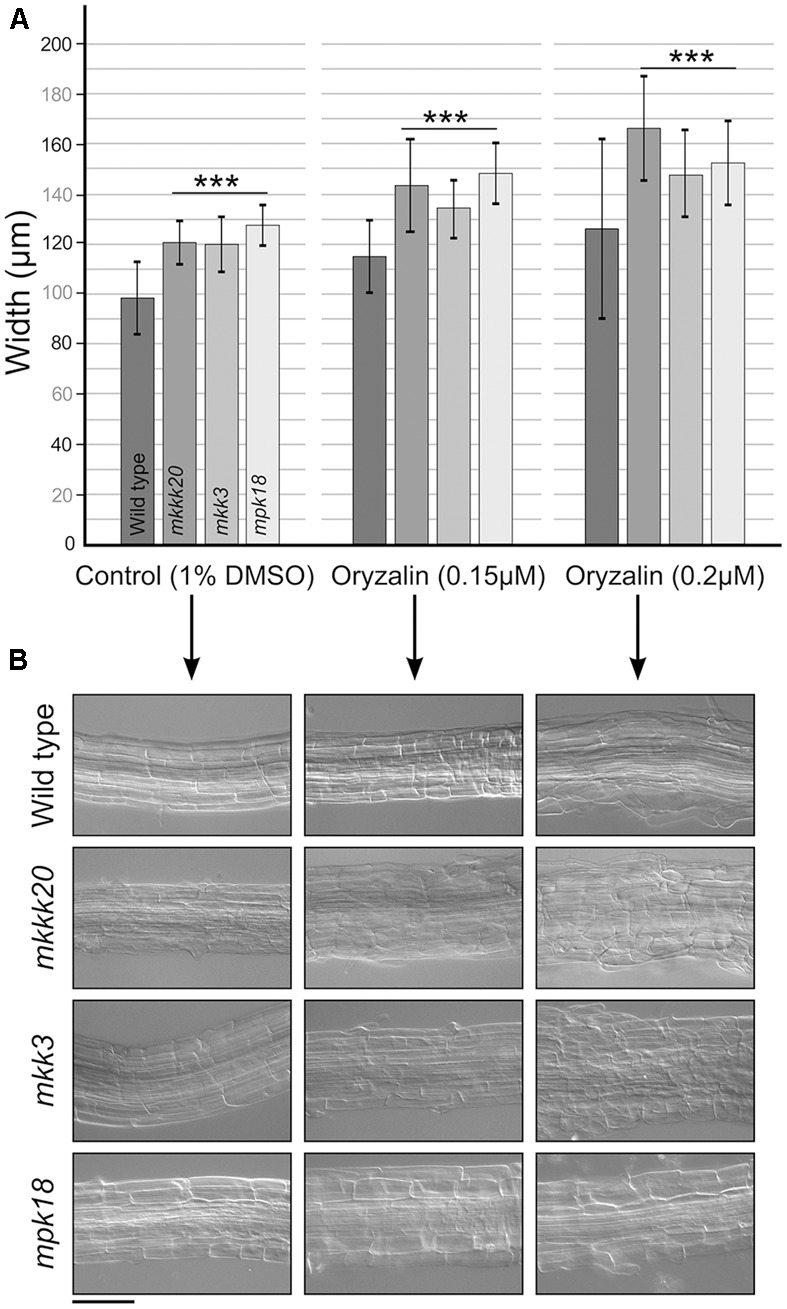
Cell width at the root elongation zone. **(A)** Width of the root elongation zone. Width was calculated from three elongation zone position (beginning, middle, end) and reported as a mean (*n* ≥ 15). **(B)** Representative observation for each single mutant in **(A)**. Data represent mean ± SD. Scale bar: 100 μm. Student’s *t*-test was used to calculate the *p*-value. Highly significant differences ^∗∗∗^*p* < 0.001.

Low doses of the microtubule-disrupting inhibitor oryzalin on growing roots are also known to generate a left-handed twisting of the roots (when viewed from above the plates), and both root skewing and root elongation are generally linked to microtubule organization and stability (Baskin et al., 1994; Thitamadee et al., 2002). Since it had been reported that *mpk18* mutant seedlings showed defects in microtubule-related functions (Walia et al., 2009), we next investigated whether *MKKK20* and *MKK3* were also involved in the same biological process. To do so, T-DNA insertional lines for the three designated kinases *mkkk20-1*, *mkkk20-2*, *mkk3-1*, and *mpk18-1* we used. Both *mkkk20* T-DNA insertion lines as well as *mkk3-1* and *mpk18-1* were considered null alleles since no mRNA could be amplified by RT-PCR analyses (**Supplementary Figure S3**). All mutant plants were morphologically analyzed and compared to *Arabidopsis* wild type plants and no obvious defect was observed at any developmental stages under normal growth conditions. However, seedling roots from *mkkk20*, *mkk3*, and *mpk18* plants were significantly shorter (*p* < 0.001) than wild type plants for all oryzalin concentration used (**Figures 6A,B**). The same phenotype was observed for *mkkk20-1* and *mkkk20-2* (**Supplementary Figure S4**; in subsequent experiments only *mkkk20-1* is used). Since *mkkk20*, *mkk3*, and *mpk18* single mutants showed defects in microtubule organization, analyses of double and triple mutants were undertaken to further explore the relationship between these kinases and microtubule functionality. When all single mutants were reciprocally crossed to each other, only the *mkkk20/mkk3* double mutant offspring were unviable. The other two double mutant pairs, *mkkk20*/*mpk18* and *mkk3/mpk18*, were fully viable. Both double mutants displayed significantly shorter roots compared to all single mutants in the presence of 0.12 and 0.15 μM oryzalin. At the highest oryzalin concentration tested (0.2 μM), no significant differences between single and double mutants was observed. Interestingly, root length was affected in the *mkk3/mpk18* double mutant in the absence of oryzalin (**Figures 6A,B**). Since obvious left-handed root twisting was observed (**Figure 6A**), root skewing angle was also measured. At 0.2 μM oryzalin concentration, all single mutants showed leftward skewing (**Supplementary Figure S5**), as observed previously with the *mpk18* mutants (Walia et al., 2009). Functional complementation of *mkkk20* and *mkk3* plants was accomplished by plant transformation through agro-infiltration with constructs encoding C-terminal His-tagged proteins (p35S::*MKKK20*-His and p35S::*MKK3*-His), in their respective mutant plants. Overexpression of the two kinases was verified by western blots in transformed plants with anti-His antibodies (**Figures 6C,D**). Complementation of single *mkkk20* and *mkk3* mutants with their respective constructs was fully restored as root length from both complemented mutants regained the WT phenotype under 0.2 μM oryzalin treatments (**Figure 6E**). Close observations of cell shape also showed that at the root elongation zone width was significantly higher than WT for the three single mutants (*p* < 0.001) at the two oryzalin concentrations used (**Figure 7A**). Elongation zone shape for each mutant is illustrated in **Figure 7B**.

## Discussion

Among the 21 *Arabidopsis* MEKKs, MKKK19, -20, and -21 form a highly supported clade within the MEKK family. Paralogous MAPKKKs of this clade have been initially studied in Solanaceous species where they were coined Fertilization-Related Kinases (FRKs) from the first three characterized members in *Solanum chacoense* (*ScFRK1*, -*2*, and -*3*) (Gray-Mitsumune et al., 2006; O’Brien et al., 2007; Daigle and Matton, 2015; Lafleur et al., 2015). This clade has considerably expanded in the Solanaceae family that includes potato and tomato (Daigle and Matton, 2015). In these two species, the FRK clade represents around 40% of all MEKKs (15 and 17 FRKs out of 36 and 39 MEKKs, respectively), compared to 14% (3 out of 21) in *A. thaliana*. The FRK class is further subdivided in four groups. *Arabidopsis* MKKK19, -20, and -21 belong to group 4, the most ancient group in dicots, while the Solanaceae family encompass the four groups. While the role of these three *Arabidopsis* kinases is largely unknown, except for the role of MKKK20 in osmotic stress (Kim et al., 2012) and in abscisic acid responses (Li et al., 2017), the three characterized Solanaceous FRKs show a clear involvement in male and female reproductive development. Here, we employed genetic and biochemical tools to study the biological significance of the *A. thaliana* *MKKK20*. Surprisingly, although *MKKK20* is strongly expressed in pollen (**Figure 3**), no obvious reproductive phenotype was observed in the loss-of-function mutant. Instead, *MKKK20* single mutants showed a clear defect in MT functions.

The Y2H screen for MKKK20 interacting partners revealed a strong and robust association with MPK18, a result that suggested the operation of an atypical MAPK pathway that bypasses MKKs (**Figure 1E**, Y2H and **Figure 2C**, BiFC). Other MKKKs have been shown to interact with proteins other than MKKs. For example, *At*MEKK1 not only interact with, and phosphorylate the transcription factor WRKY53, but can also bind to the promoter region of the WRKY53 gene to increase its relative expression (Miao et al., 2007). Although not considered a MEKK, the Raf-like Constitutive Triple Response1 MKKK (CTR1) interacts with and directly phosphorylates Ethylene-insensitive 2 (EIN2), which is not a MKK (Ju et al., 2012). In alfalfa, the MKKK OMTK1 (oxidative stress-activated MAP triple-kinase 1), which is activated by hydrogen peroxide, interacts directly with the MMK3 MAPK, thus bypassing the need for an intermediate MKK (Nakagami et al., 2004). Since MAPK cascades are prototypically activated by three-tiered sequential phosphorylation events, the involvement of a MKK in this putative cascade was addressed. A directed pairwise Y2H assay revealed that, among the 10 AtMKKs, MKK3 showed by far the strongest interaction with MKKK20. However, interaction between MKK3 and MPK18 in two-hybrid assays proved to be very weak compared to the strong MKKK20–MKK3 interaction (**Figure 1C**). An earlier directed two-hybrid screen between all *Arabidopsis* MKKs and MPKs, also showed no interaction between MKK3 and MPK18 (Lee et al., 2008), and the weak interaction observed between MKK3 and MPK18 (**Figure 1E**) was not validated by the BiFC assay (**Figures 2E,F**) nor by a phosphorylation assay (**Figure 5D**).

The first results from kinase activity assays demonstrated that MKKK20 autophosphorylates strongly (**Figures 5A–D**). Since eukaryotic protein kinases (EPKs) catalytic activity depends on phosphorylation of their activation loop, leading to a conversion from inactive to active conformation, the activation loop status of the MKKK20 expressed in bacteria was investigated (Bossemeyer, 1995; Nolen et al., 2004). Interestingly, autophosphorylation of EPKs seemed to be shared by most eukaryotic protein kinases (Beenstock et al., 2016). LC-MS/MS results showed that, inside the activation segment, bordered by the DFG motif in subdomain VII and the APE motif in subdomain VIII, two residues from the MKKK20 activation loop (threonine^161^ and serine^166^) were phosphorylated (**Supplementary Figure S2**). Such phosphorylation is important for MAPKKK activation as demonstrated for human Mixed Lineage Kinase-3 (Leung and Lassam, 2001). In addition, MKKK20 phosphorylates both MKK3 and MPK18 equally, whereas no phosphorylation was observed when MKK3 and MPK18 are incubated together (**Figure 5D**). When all three kinases were present, MKKK20 phosphorylated equally MKK3 and MPK18. The presence of a phosphorylated MKK3 did not result in any obvious increase in MPK18 phosphorylation. Thus, the evidence suggests that MPK18 is not a *bona fide* MKK3 target, and that the pattern of activation of these kinases is an exception to the canonical MAPK signaling pathway. As noted above, this is not without precedent. Indeed, three characterized *Nicotiana* MAPKKKs (*Nb*MAPKKKα, *Nb*MAPKKKβ, and *Nb*MAPKKKγ) were found to form a linear cascade leading to programmed cell death in tobacco cells (Hashimoto et al., 2012). On the other hand, our two-hybrid analysis and kinase assays demonstrated that MKKK20 interacts with and phosphorylates both MKK3 and MPK18.

*Arabidopsis* MPK18 had previously been shown to mediate cortical microtubule functions (Walia et al., 2009). Loss-of-function mutation in *MPK18* results in more stable microtubule arrays and *mpk18* roots display more abundant and better co-aligned microtubule polymers in their elongation zones as a result of MT dynamic instability problems (Walia et al., 2009). When treated with oryzalin, the *mkkk20* mutant displayed significantly diminished seedling root length compared to the similarly treated wild type plants (**Figures 6A,B**). This phenotype is similar to the one observed in *mpk18* plants, suggesting that both MKKK20 and MPK18 could be participating in a MAPK signaling pathway that mediates cortical microtubule function. In addition, *mkk3* mutant roots, like *mkkk20* and *mpk18*, were significantly shortened when treated with oryzalin, suggesting that these three kinases could be involved in a classical MAPK cascade. Thus, experimental evidence from both genetic and biochemical studies suggested the involvement of MKKK20, MKK3, and MPK18 in cortical microtubule functions in *Arabidopsis* cells, but in more than one signaling cascade. Since no protein–protein interaction or phosphorylation was observed between MKK3 and MPK18, this suggests that these three kinases act through two independent pathways involved in MT functions. The first would represent a canonical MAPK cascade, consisting of MKKK20, MKK3, and an as yet-unknown MAPK, while the second would be a non-canonical MAPK cascade with MKKK20 and MPK18 interacting directly, thus bypassing the need for a MKK intermediary. MKKK20 would therefore activate MKK3, which could in turn target downstream MAPKs (other than MPK18) involved in microtubule functions. Alternatively, MKKK20 could directly interact with MPK18 that would activate other target(s) (e.g., MAPs), modulating microtubule functions. Such direct MPK18 activation through phosphorylation of its activation loop TDY motif was validated using an anti-p-ERK antibody that specifically targets the [T-X-Y] MPK18 activation loop (**Figure 5E**). Interestingly, another D-type member, MPK9, was also shown to be activated through an independent MAPK cascade manner (Nagy et al., 2015).

Regarding the first pathway, two MPKs involved in MT functions had been previously found to interact with MKK3: MPK4 and MPK6 (Popescu et al., 2009). MPK4 acts downstream from two MKKKs (ANP1-2; also known as MKKK1 and MKKK2) involved in microtubule organization (Beck et al., 2010, 2011). In addition, MPK4 phosphorylates the microtubule-associated proteins *At*MAP65-1 and *At*MAP65-2, which are two structural components of microtubule arrays involved in cell division (Sasabe et al., 2011), and possess canonical serine/threonine-proline MAPK phosphorylation sites. MPK6 has been functionally linked to MTs since it localizes to the plasma membrane and is involved in root development and cell division control (Muller et al., 2010). MPK6 has been also shown to interact with γ-tubulin and the MT plus end protein EB1 (Kohoutova et al., 2015). Thus, both MPK4 and MPK6 represent strong candidates for completing the proposed MKKK20/MKK3 module. It is worth mentioning that MKK3 plays an essential role in the activation of MPK6 through the phytohormone jasmonic acid (JA; Takahashi et al., 2007). In a large microarray transcriptional analysis of several MAPK signaling genes, jasmonates were also found to induce transcription of *MKKK20* (Menges et al., 2008). Thus, under the influence of JA, MKKK20 could potentially activate the MKK3/MPK6 cascade thereby affecting MT function. Interestingly, JA treatment induces changes in the orientation of cortical MTs during potato tuberization and cell expansion, consistent with the idea that JA might control the direction of cell expansion by changing the arrangement of MTs (Koda, 1997; Cenzano et al., 2003). Recently, a MKKK20-MKK5-MPK6 cascade was also shown to modulate abscisic acid responses, regulating cell division and cell elongation mainly at the root elongation zone (Li et al., 2017). As for MKK5, MKK4 was also shown to interact with MKKK20 in a BiFC assay. Surprisingly, previous results from the *Brassica napus* MKKK20 ortholog (BnaMKKK20; Sun et al., 2014), showed no interaction between BnaMKKK20 and BnaMKK4 or BnaMKK5, a result also observed in our Y2H assay with the *A. thaliana* MKKK20 (**Figure 1**). Furthermore, MKKK20 from *A. thaliana* (**Figure 1**) and *B. napus* (Sun et al., 2014) strongly interacted with MKK3, in both Y2H and BiFC assays. It is noteworthy to mention that in this case, different BiFC assay systems (microparticle bombardment vs *Agrobacterium* infiltration) were used. The MKKK20–MKK3 Y2H interaction was also recently mentioned in (Colcombet et al., 2016), emphasizing the strong interaction between MKKK20 and MKK3. Taken together, the short root phenotype observed in *mkkk20* plants could result in overlapping functions of MKKK20 in microtubule organization and ABA responses. Interestingly, it had been demonstrated that ABA modulates microtubule organization and stability (Sakiyama and Shibaoka, 1990; Sakiyama-Sogo and Shibaoka, 1993; Takatani et al., 2015). Likewise, the same MPK cascade elements can be involved in different biological contexts (Meszaros et al., 2007; Colcombet et al., 2016). Our results indicate that MKKK20 would act in separate cascades in response to different stimuli.

Overall, our results showed that MKKK20 plays a role in plant root microtubule functions, thereby affecting root growth and development. Two distinct processes regulate plant organ growth; production of cells and expansion. Root growth rate is determined primarily by expansion, yet is also influenced by cell division and production (Beemster and Baskin, 1998). Indeed, it has been reported that the growth of *Arabidopsis* roots was accompanied by increased cell production leading to cell elongation (Baskin et al., 1995). Cortical as well as mitotic microtubules play an essential role in both elongation and division, and a wide range of MAPs (microtubule-associated proteins) have been associated with these developmental stages in plant cells (Hamada, 2014). It has been shown that the phosphorylation state of MAPs influences microtubule stability (Drechsel et al., 1992; Mandelkow et al., 1995), and phosphorylation of MAPs leads to reduced binding to the microtubule cytoskeleton due to a reduction in MAP-microtubule affinity (Drewes et al., 1998). Our results are consistent with the idea that these potentially convergent phosphorylation-based signaling pathways play a role in controlling microtubule dynamics. The binding of specific MAPs to MTs results in structural regulation via interconnected polymerization/catastrophe events collectively referred to as dynamic instability (Hamada, 2014). Some of these MAPs play crucial roles in microtubule orientation or anisotropy. This could be explained by a model in which some structural MAPs (downstream of the canonical and non-canonical MKKK20 cascades) would be lesser phosphorylated in the absence of MKKK20, resulting in their higher affinity for MT polymer chains (Nogales, 2000), and perturbation of the normal array alignment. Therefore, such perturbation could affect directly the normal growth of the plant cell wall, cortical microtubule functions being disturbed, as observed in the elongation zone (**Figure 7**). The two MKKK20 pathways, one that includes MPK18, and the other MKK3 (possibly with MPK4 or MPK6), are likely to target different MAPs and exert different influences on MT dynamics and organization. Indeed, the phenotype of *mpk18* is not as dramatic as that of *mpk4* (Komis et al., 2011) and MPK4 was found to co-localize with microtubules, unlike MPK18 (Walia et al., 2009). Moreover, inhibition of phosphorylation via the *mkk3* mutation is likely to cause MT hyperstabilization, as seen with *mpk18-1* mutant (Walia et al., 2009). The summation of different pathway disturbances in the *mkkk20* mutant could therefore lead to more drastic perturbation of microtubule dynamics. This is supported by the observation that, in the absence of oryzalin, the *mkk3/mpk18* double mutant root length is significantly shorter than in all other lines (**Figures 6A,B**). Our understanding of the role played by plant MAPK signaling in regulating microtubule functions still needs to be refined. The involvement of multiple MAPK members in numerous biological processes often compromises a clear understanding of phenotypic specificity. Full characterization of the signaling network, including cross talk between parallel cascades, will ultimately be needed to develop a coherent biological picture.

## Author Contributions

RB, FB, SD, and AL-H carried out the experiments. BE provided study materials and critical revision of the manuscript. RB and DM designed most experiments and wrote the manuscript.

## Conflict of Interest Statement

The authors declare that the research was conducted in the absence of any commercial or financial relationships that could be construed as a potential conflict of interest.

## Footnotes

1 http://www.arabidopsis.org

2 http://www.addgene.org/13668/

3 http://www.addgene.org/11520/

4 https://genevestigator.com/gv

5 http://bbc.botany.utoronto.ca

6 http://suba.live

## Supplementary Material

FIGURE S1 Kinase expression patterns assessed by RT-PCR. RT-PCR analysis of *MKKK20*, *MKK3*, and *MPK18* gene expression in various organs of *Arabidopsis thaliana*. The actin2 gene was used as an internal control for equal loading. Specific primers for each gene can be found in **Supplementary Table S3**. Twenty-four cycles were performed for *MKKK20*, *MKK3*, and *MPK18*, while 20 cycles were used for *Act2*.

FIGURE S2 MKKK20 autophosphorylation in bacteria. LC-MS/MS MKKK20 phosphosites analysis from WT and kinase dead protein expressed in bacteria. His-Tag MKKK20/WT and MKKK20-KD proteins expressed in bacteria were separated on SDS-PAGE gel. MKKK20/WT high and low molecular weight bands as well as MKKK20-KD were excised and sent for mass spectrometry analyses. Phosphorylated serine (S), threonine (T), and tyrosine (Y) are shown in red, blue, and green, respectively. Full gray bar represents the coverage of the phosphorylated peptides from the four MKKK20/WT experiments. Dashed gray bar represent an extra single phosphorylated peptide found in MKKK20-LMW-1. Black bar represents the overall peptide coverage observed for all MKKK20 proteins. Asterisks indicate the six phosphorylated Ser, Thr, or Tyr that are unique to HMW or LMW MKKK20/WT. For identification of MS/MS peptides, see **Supplementary Table S7**.

FIGURE S3 RT-PCR showing lack of *MKKK20-1*, *MKKK20-2*, *MKK3*, and *MPK18* transcript in 10-day-old seedlings from each T-DNA mutant. RT-PCR product from five different seedlings are shown for *mkkk20*-1, -2 **(A)**, *mkk3* **(B)**, and *mpk18* **(C)** side by side with wild type using specific primers corresponding to each of the three genes. No amplification was observed with cDNA from *mkkk20*-1, -2 likewise, no expression was seen for *mkk3* and *mpk18* after 32 cycles for each gene. The same number of cycle was as used for the *actin2* as an internal control. All gene-specific primers are listed on **Supplementary Table S2**.

FIGURE S4 *MKKK20* T-DNA insertional mutant phenotype. Root length **(A)** and skewing angle **(B)** of 8-day-old single *mkkk20-1* and *mkkk20-2* mutant seedlings grown on DMSO and with various concentrations of oryzalin. Both *MKKK20* T-DNA mutant lines showed identical behavior.

FIGURE S5 Mutants root skewing angle. The root skewing angle from 6-day-old WT, *mkkk20*-1, *mpk18*, *mkk3*, *mkkk20/mpk18*, and *mkk3/mpk18* were measured. Oryzalin (0.2 μM) treatment induced significantly lower root-skewing angle in all mutants comparing to the wild type. Results are expressed as means ± SD (*n* ≥ 28). Three distinct groups were calculated a, b, and c based on one-way ANOVA multiple comparison with Tukey’s test, ^∗∗∗^*p* < 0.001.

TABLE S1 T-DNA insertion lines.

TABLE S2 Primers used for T-DNA lines genotyping.

TABLE S3 Primers used for promoter-GUS constructs and RT-PCR analyses.

TABLE S4 Primers used for protein expression constructs.

TABLE S5 Primers used for *mkkk20* and *mkk3* complementation constructs.

TABLE S6 Gene ontology analysis of MKKK20 interacting proteins.

TABLE S7 LC-MS/MS peptide sequencing data.
