# Supplementary figures and images for: The Arabidopsis Mitogen-Activated Protein Kinase Kinase Kinase 20 (MKKK20) Acts Upstream of MKK3 and MPK18 in Two Separate Signaling Pathways Involved in Root Microtubule Functions
Source: Front Plant Sci. 2017 Aug 8;8:1352. doi: 10.3389/fpls.2017.01352 (PMC5550695; doi:10.3389/fpls.2017.01352)

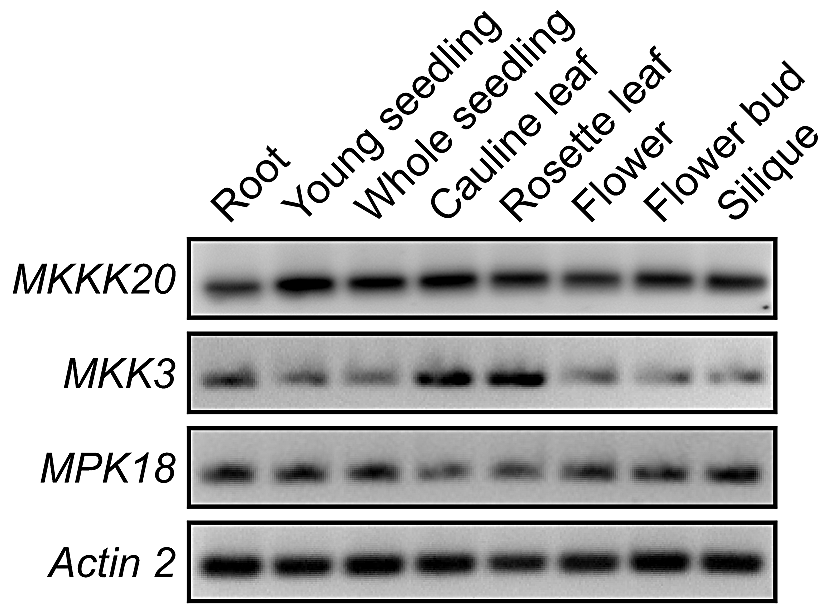

Supplement: FIGURE S1 — Kinase expression patterns assessed by RT-PCR. RT-PCR analysis of MKKK20, MKK3, and MPK18 gene expression in various organs of Arabidopsis thaliana. The actin2 gene was used as an internal control for equal loading. Specific primers for each gene can be found in Supplementary Table S3. Twenty-four cycles were performed for MKKK20, MKK3, and MPK18, while 20 cycles were used for Act2. [file Supplementary_Figure_1.TIF]

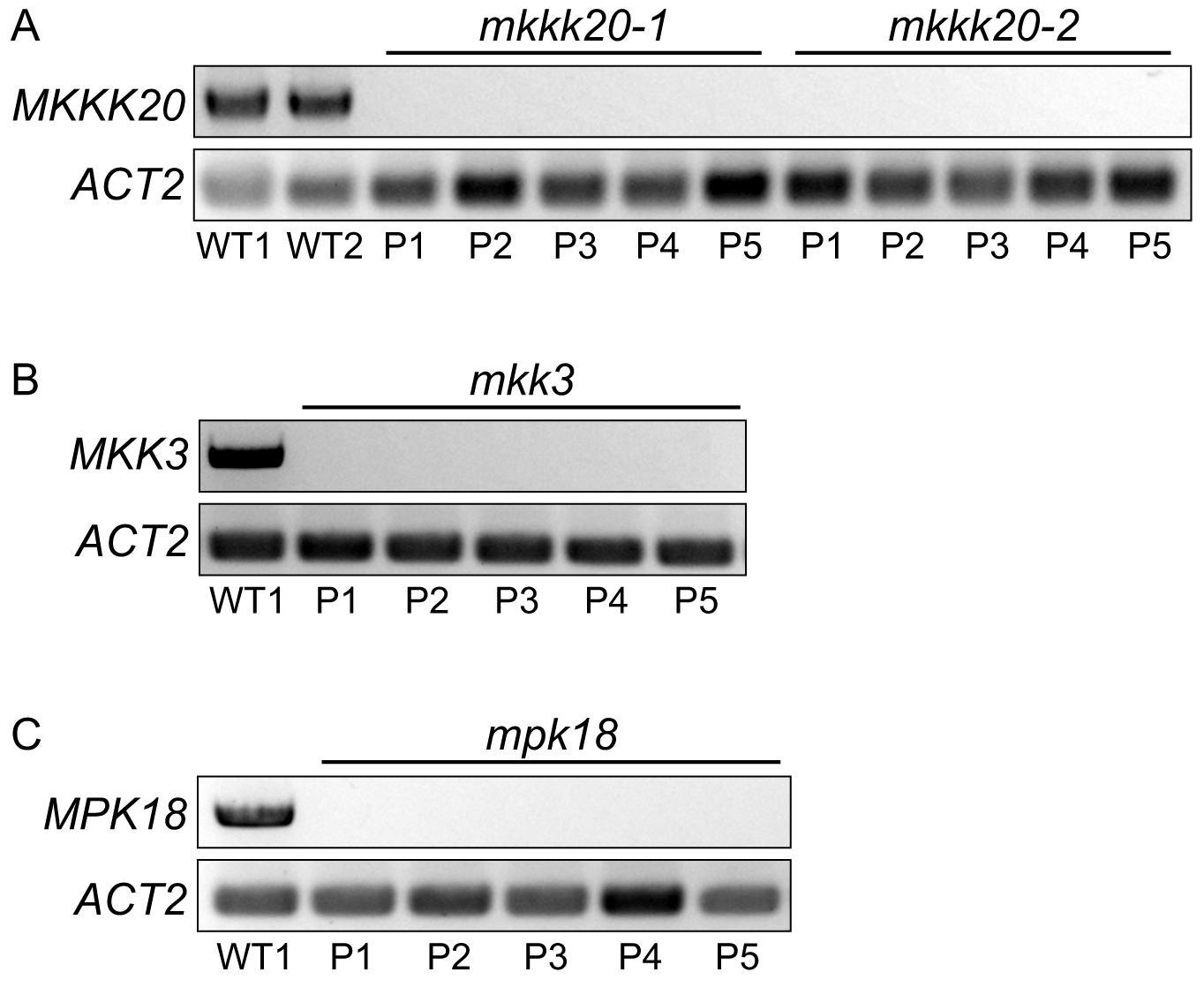

Supplement: FIGURE S3 — RT-PCR showing lack of MKKK20-1, MKKK20-2, MKK3, and MPK18 transcript in 10-day-old seedlings from each T-DNA mutant. RT-PCR product from five different seedlings are shown for mkkk20-1, -2 (A), mkk3 (B), and mpk18 (C) side by side with wild type using specific primers corresponding to each of the three genes. No amplification was observed with cDNA from mkkk20-1, -2 likewise, no expression was seen for mkk3 and mpk18 after 32 cycles for each gene. The same number of cycle was as used for the actin2 as an internal control. All gene-specific primers are listed on Supplementary Table S2. [file Supplementary_Figure_3.TIF]

A

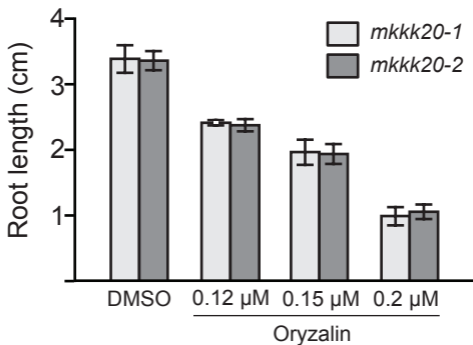

B

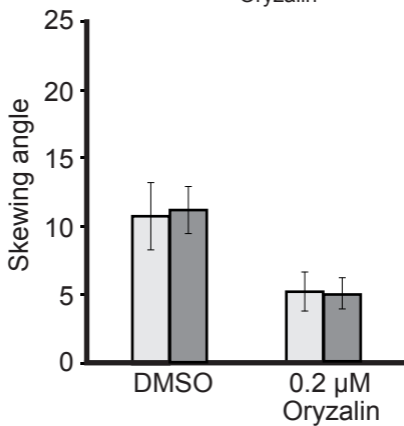

Supplement: FIGURE S4 — MKKK20 T-DNA insertional mutant phenotype. Root length (A) and skewing angle (B) of 8-day-old single mkkk20-1 and mkkk20-2 mutant seedlings grown on DMSO and with various concentrations of oryzalin. Both MKKK20 T-DNA mutant lines showed identical behavior. [file Supplementary_Figure_4.PDF]

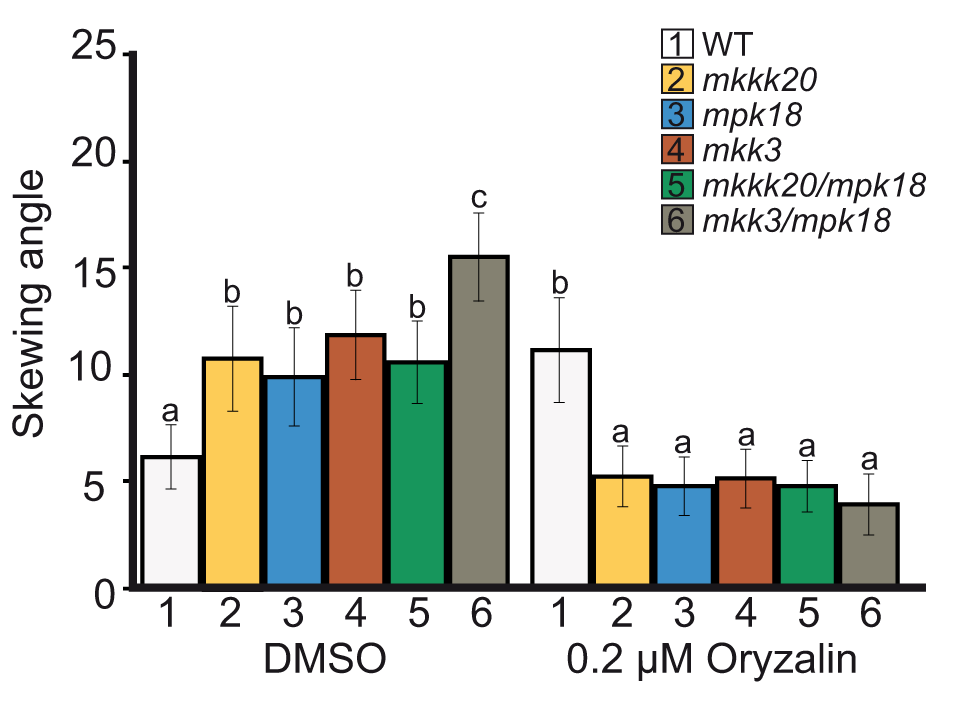

Supplement: FIGURE S5 — Mutants root skewing angle. The root skewing angle from 6-day-old WT, mkkk20-1, mpk18, mkk3, mkkk20/mpk18, and mkk3/mpk18 were measured. Oryzalin (0.2 μM) treatment induced significantly lower root-skewing angle in all mutants comparing to the wild type. Results are expressed as means ± SD (n ≥ 28). Three distinct groups were calculated a, b, and c based on one-way ANOVA multiple comparison with Tukey’s test, ∗∗∗p < 0.001. [file Supplementary_Figure_5.TIF]
