# Supplementary material for: The Arabidopsis Mitogen-Activated Protein Kinase Kinase Kinase 20 (MKKK20) Acts Upstream of MKK3 and MPK18 in Two Separate Signaling Pathways Involved in Root Microtubule Functions
Source: Front Plant Sci. 2017 Aug 8;8:1352. doi: 10.3389/fpls.2017.01352 (PMC5550695; doi:10.3389/fpls.2017.01352)
Supplement: FIGURE S2 — MKKK20 autophosphorylation in bacteria. LC-MS/MS MKKK20 phosphosites analysis from WT and kinase dead protein expressed in bacteria. His-Tag MKKK20/WT and MKKK20-KD proteins expressed in bacteria were separated on SDS-PAGE gel. MKKK20/WT high and low molecular weight bands as well as MKKK20-KD were excised and sent for mass spectrometry analyses. Phosphorylated serine (S), threonine (T), and tyrosine (Y) are shown in red, blue, and green, respectively. Full gray bar represents the coverage of the phosphorylated peptides from the four MKKK20/WT experiments. Dashed gray bar represent an extra single phosphorylated peptide found in MKKK20-LMW-1. Black bar represents the overall peptide coverage observed for all MKKK20 proteins. Asterisks indicate the six phosphorylated Ser, Thr, or Tyr that are unique to HMW or LMW MKKK20/WT. For identification of MS/MS peptides, see Supplementary Table S7. [file Supplementary_Figure_2.PDF]

```
MKKK20-HMW-1   MEWVRGETIGFGTFSTVSTATKSRNSGDFPALIAVKSTDAYGAASLSNEKSVLDSLGDCPEIIRCYGEDSTVENGEEMHNLLEYASRGSLASYMKKLGG   100
MKKK20-HMW-2   MEWVRGETIGFGTFSTVSTATKSRNSGDFPALIAVKSTDAYGAASLSNEKSVLDSLGDCPEIIRCYGEDSTVENGEEMHNLLEYASRGSLASYMKKLGG   100
MKKK20-LMW-1   MEWVRGETIGFGTFSTVSTATKSRNSGDFPALIAVKSTDAYGAASLSNEKSVLDSLGDCPEIIRCYGEDSTVENGEEMHNLLEYASRGSLASYMKKLGG   100
MKKK20-LMW-2   MEWVRGETIGFGTFSTVSTATKSRNSGDFPALIAVKSTDAYGAASLSNEKSVLDSLGDCPEIIRCYGEDSTVENGEEMHNLLEYASRGSLASYMKKLGG   100
MKKK20-KD      MEWVRGETIGFGTFSTVSTATKSRNSGDFPALIAVMSTDAYGAASLSNEKSVLDSLGDCPEIIRCYGEDSTVENGEEMHNLLEYASRGSLASYMKKLGG   100

MKKK20-HMW-1   EGLPESTVRRHTGSVLRGLRHIHAKGFAHCDIKLANILLFNDGSVKIADFGLAMRVDGDLTALRKSVEIRGTPLYMAPECVNDNEYGSAADVWALGCAVV   200
MKKK20-HMW-2   EGLPESTVRRHTGSVLRGLRHIHAKGFAHCDIKLANILLFNDGSVKIADFGLAMRVDGDLTALRKSVEIRGTPLYMAPECVNDNEYGSAADVWALGCAVV   200
MKKK20-LMW-1   EGLPESTVRRHTGSVLRGLRHIHAKGFAHCDIKLANILLFNDGSVKIADFGLAMRVDGDLTALRKSVEIRGTPLYMAPECVNDNEYGSAADVWALGCAVV   200
MKKK20-LMW-2   EGLPESTVRRHTGSVLRGLRHIHAKGFAHCDIKLANILLFNDGSVKIADFGLAMRVDGDLTALRKSVEIRGTPLYMAPECVNDNEYGSAADVWALGCAVV   200
MKKK20-KD      EGLPESTVRRHTGSVLRGLRHIHAKGFAHCDIKLANILLFNDGSVKIADFGLAMRVDGDLTALRKSVEIRGTPLYMAPECVNDNEYGSAADVWALGCAVV   200

MKKK20-HMW-1   EMFSGKTAWSVKEGSHFMSLLIRIGVGDELPKIPEMLSEEGKDFLSKCFVKDPAKRWTAEMLLNHSFVTIDLEDDHRENFVVKVKDEDKVLMSPKCPFEF   300
MKKK20-HMW-2   EMFSGKTAWSVKEGSHFMSLLIRIGVGDELPKIPEMLSEEGKDFLSKCFVKDPAKRWTAEMLLNHSFVTIDLEDDHRENFVVKVKDEDKVLMSPKCPFEF   300
MKKK20-LMW-1   EMFSGKTAWSVKEGSHFMSLLIRIGVGDELPKIPEMLSEEGKDFLSKCFVKDPAKRWTAEMLLNHSFVTIDLEDDHRENFVVKVKDEDKVLMSPKCPFEF   300
MKKK20-LMW-2   EMFSGKTAWSVKEGSHFMSLLIRIGVGDELPKIPEMLSEEGKDFLSKCFVKDPAKRWTAEMLLNHSFVTIDLEDDHRENFVVKVKDEDKVLMSPKCPFEF   300
MKKK20-KD      EMFSGKTAWSVKEGSHFMSLLIRIGVGDELPKIPEMLSEEGKDFLSKCFVKDPAKRWTAEMLLNHSFVTIDLEDDHRENFVVKVKDEDKVLMSPKCPFEF   300

MKKK20-HMW     DDWDSFTLDSNPSFDSPVERLGSLVSGSIPDWSVGGSWLTVR   342
MKKK20-LMW     DDWDSFTLDSNPSFDSPVERLGSLVSGSIPDWSVGGSWLTVR   342
MKKK20-HMW     DDWDSFTLDSNPSFDSPVERLGSLVSGSIPDWSVGGSWLTVR   342
MKKK20-LMW     DDWDSFTLDSNPSFDSPVERLGSLVSGSIPDWSVGGSWLTVR   342
MKKK20-KD      DDWDSFTLDSNPSFDSPVERLGSLVSGSIPDWSVGGSWLTVR   342
```
